# The Effects of Neuroinflammation Induced by Typhoid Vaccine on Resting and Task‐Based Electroencephalography

**DOI:** 10.1002/brb3.70249

**Published:** 2024-12-31

**Authors:** Julia R Plank, Joseph CC Chen, Frederick Sundram, Nicholas Hoeh, Suresh Muthukumaraswamy, Joanne C Lin

**Affiliations:** ^1^ Faculty of Medical and Health Sciences, School of Pharmacy University of Auckland Grafton Auckland New Zealand; ^2^ Faculty of Medical and Health Sciences, Department of Psychological Medicine, School of Medicine University of Auckland Grafton Auckland New Zealand

**Keywords:** attention, electroencephalography, neuroinflammation, neurophysiology

## Abstract

**Introduction:**

Considerable evidence suggests a pathophysiological role of neuroinflammation in psychiatric disorders. Lumbar puncture and positron emission tomography (PET) show increased levels of inflammation in psychiatric disorders. However, the invasive nature of these techniques, as well as their expense, make them undesirable for routine use in patients. Electroencephalography (EEG) is noninvasive, affordable and shows potential as a clinical tool for detection of neuroinflammation.

**Methods:**

In this randomized, crossover design, placebo‐controlled, double‐blind study, typhoid vaccine was administered to 20 healthy volunteers to induce a low level of neuroinflammation. EEG was recorded before and after placebo/vaccine administration during resting‐state and during performance of the Attention Network Test (ANT). Resting‐state EEG was analyzed using spectral power analysis, and time–frequency analysis was used for the EEG from the ANT. Behavioral data were assessed using linear mixed models and Spearman's correlations.

**Results:**

Behavioral results from the ANT showed no decrement in performance following the vaccine, consistent with previous studies. During eyes‐open resting, there was a relative decrease in right‐frontal delta power in the vaccine condition compared to placebo. There was a trend toward greater alpha power suppression in the alerting response of the attentional network; however, this finding did not reach significance.

**Conclusion:**

Decreased resting‐state delta power may reflect an unpleasant internal state conferred by the vaccine. Inflammation did not significantly affect attention networks. The absence of significant alterations may be due to an insufficient inflammatory response. Further studies are needed to assess the feasibility of EEG as a technique for detection of neuroinflammation.

## Graphical abstract

• Inflammation induced by typhoid vaccine did not significantly affect attention networks, despite a trend toward increased alerting‐related alpha suppression. •A relative decrease was observed in right‐frontal delta power during eyes‐open in vaccine versus placebo. •Behavioral results showed no decrement in performance in the inflammation condition.

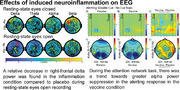

## 1 Introduction

Accumulating evidence points to a critical role of neuroinflammation in the pathophysiology of psychiatric disorders. Positron emission tomography (PET), lumbar puncture, postmortem examination, and peripheral immune markers have identified signs of neuroinflammation in major depressive disorder, bipolar disorder, schizophrenia, and other psychiatric conditions (Najjar et al. 2013). Neuroinflammation may represent a new treatment target with the potential for developing more efficacious treatments. However, this approach is heavily constrained by the lack of a noninvasive tool for measurement of neuroinflammation in clinical populations.

Electrical activity measured by EEG contains oscillatory activity, which can be classified by frequency band (e.g., delta 2–4 Hz). Changes in oscillatory activity are observed in a variety of neurological and psychiatric conditions including epilepsy and major depressive disorder (Debener et al. 2000; Seneviratne et al. 2013). EEG can be used to locate areas of brain damage (e.g., through focal slowing in injured areas; Nuwer et al. 2005), manage anesthesia by monitoring sedation depth (e.g., through slowing of brain activity and burst suppression; Sun et al. 2020), and test cognitive engagement (e.g., through event‐related potentials and oscillatory activity; Curley et al. 2018). EEG is an attractive tool to use for routine clinical assessments due to its relatively low cost, easy accessibility, and the diversity of options available for analysis. EEG also offers excellent temporal resolution, though the spatial resolution of EEG is poor in comparison to techniques such as functional magnetic resonance imaging (fMRI). EEG is a feasible option for clinical practice and is already used in clinical settings, for example in the assessment of epilepsy (Seneviratne et al. 2013).

Prior studies have investigated disturbance of the EEG signal following administration of an acute inflammatory stimulus. van den Boogaard et al. (2010) monitored 15 healthy male volunteers using EEG before and after administration of lipopolysaccharide (LPS) derived from *E. coli*. Participants demonstrated a significant relationship between increased cortisol and increased occipital peak frequency. The authors suggest that the EEG indicates a higher state of alertness; possibly driven by a “fight‐or‐flight” stress hormone response due to LPS. The study demonstrated preliminary evidence of an inflammatory effect on EEG. However, the work was limited by the small sample size, which conferred relatively low statistical power (van den Boogaard et al. 2010). Further studies are needed to determine the effect of experimentally induced inflammation on EEG.

A further study of the effect of inflammation on EEG was conducted by Balter et al. (2019). Twenty healthy males received typhoid vaccine in a crossover design placebo‐controlled study. The authors aimed to understand the mechanism by which inflammation affects attentional processes. The participants completed a version of the Attention Network Test (ANT)—a popular test of three attentional processes: alerting, orienting, and executive control (Fan et al. 2002). The authors found that the level of inflammation correlated significantly with alpha power suppression. Alpha power suppression may reflect increasing cognitive load (Bazanova and Vernon 2014). Therefore, this correlation may indicate greater cognitive exertion in the presence of inflammation compared to the placebo condition. These results have not so far been replicated and additional studies are needed to confirm their findings and to further explore the potential of EEG to provide a reliable indicator of neuroinflammatory processes.

The mechanism by which neuroinflammation may affect alpha power and cognitive function should be considered. Neuroinflammation involves a cascade of reactions including activation of microglia and astrocytes, and the release of inflammatory cytokines such as interleukin (IL)‐6, tumor necrosis factor (TNF)‐α, and IL‐1β (Benarroch 2024). These inflammatory molecules can in turn disrupt synaptic signaling and neurotransmitter balance, including alterations in glutamatergic and GABAergic transmission. Alpha power suppression may reflect these disruptions. For example, inflammation may reduce inhibitory GABAergic activity or increase excitatory glutamatergic signaling, leading to hyperexcitability of neural circuits (Pardillo‐Díaz et al. 2022). Given that alpha oscillations may reflect selective attention mechanisms driven by top‐down demands (Foxe and Snyder 2011), a suppression of alpha power may reflect a disrupted ability in regulating information flow, that is, reduced efficiency in prioritizing relevant signals and suppressing distractions during cognitive tasks. Furthermore, inflammation‐induced oxidative stress and mitochondrial dysfunction may impair metabolic processes (Pardillo‐Díaz et al. 2022), in turn increasing the cognitive load required to perform tasks as the brain compensates for reduced efficiency in neural communication. The observed correlation between inflammation and alpha power suppression suggests EEG may reflect these underlying biological processes; however, replication of these findings is essential to establish alpha power suppression as a potential biomarker for neuroinflammatory states.

The present study aimed to replicate the effect of neuroinflammation on the EEG signal during the ANT. Typhoid vaccine was administered to participants to induce a temporary, low level of neuroinflammation in a crossover design, placebo‐controlled study. In addition to the ANT, a resting‐state EEG was recorded as previous animal studies found alterations to spectral power following administration of an acute inflammatory stimulus (Schiffelholz and Lancel 2001; Lancel et al. 1995; Ingiosi and Opp 2016; Albrecht et al. 2018). Male rats showed elevated delta and theta 6 h after LPS administration (Albrecht et al. 2018). Increases in low‐frequency delta power are often observed during states of unconsciousness or deep sleep (Knyazev 2012; Hlinka et al. 2010). The observed increases in delta power were linked to lethargy and symptoms of sickness in the rats. Given endotoxin may also induce fatigue in human subjects (Schedlowski, Engler, and Grigoleit 2014), we expected to find reduced delta power in our study. Vaccine‐induced modification of the resting‐state EEG in human participants could represent a useful biomarker of low‐level inflammatory processes with potential applications to clinical populations.

## 2 Materials and Methods

### 2.1 Study Design

Twenty healthy volunteers (10 males, mean age = 34 ± 7.26) participated in a randomized, placebo‐controlled, double‐blind, crossover design study as described previously (Plank et al. 2022; Plank et al. 2024). All participants gave written informed consent prior to the experiments. Participants’ resting‐state and task‐based EEG were recorded four times across two study days: preplacebo, postplacebo, prevaccine, and postvaccine. The placebo day and vaccine day were separated by at least one week. A diagram of the flow of the study is shown in **Figure** **S1**. Ethical approval for the study was granted by the Northern Health and Disability Ethics Committee (19/NTB/8) and the trial was registered at ANZCTR (ACTRN12619000738123).

### 2.2 EEG Data Acquisition

All EEG data were recorded in a Faraday cage to reduce impacts of external electrical fields. EEG was recorded at 1 kHz and 0.1 µV resolution using actiCAP‐Slim 64‐channel Ag/AgCl active shielded electrodes and BrainAmp MRPlus amplifiers (Brain Products GmbH, Germany). The online reference was FCz and ground was AFz. Electrode impedance below 10 kΩ was reached before recording. Participants were seated at approximately 80 cm distance from an “ASUS VG248QE 17” color LCD screen. Screen resolution was set at 1920×1080 with 144 Hz refresh rate. Stimuli were presented and behavioral responses were recorded using MATLAB v2020 Psychophysics toolbox (Mathworks, MA, United States).

### 2.3 Resting‐State Procedure

Prior to the ANT, participants completed two resting‐state tasks during which EEG was continuously recorded. Participants were asked to avoid eye rolling, jaw clenching, and head movements during all EEG recordings. Firstly, participants were instructed to rest with their eyes open for 5 min, while looking at a central black cross on a white background provided on the screen in front of them. Secondly, participants were instructed to rest with their eyes closed for 5 min.

### 2.4 ANT Procedure

**FIGURE 1:**
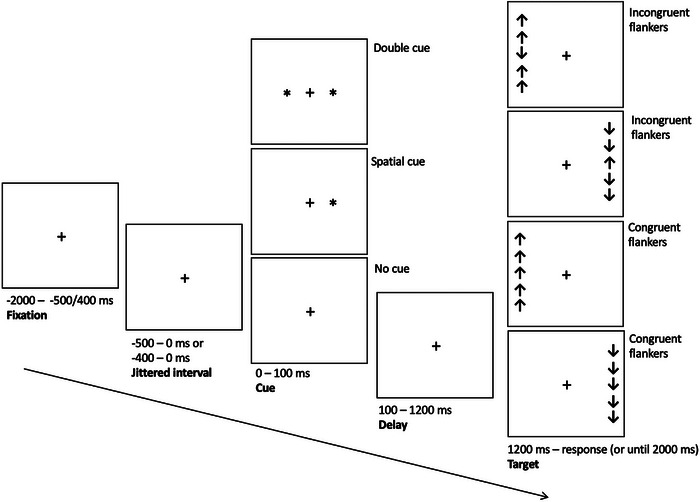
ANT schematic. Each trial begins at –2000 ms with presentation of central fixation cross which remains on screen throughout trial. A jittered interval is presented at –500 or –400 ms. Trials are time‐locked to cue onset at 0 ms. The cue condition (no cue, spatial cue, or double cue) is presented for 100 ms, followed by a delay until target array presentation at 1200 ms. The target array (incongruent or congruent flankers) is presented until the participant responds via key press or at 2000 ms. Participants were asked to report the direction of the central target stimulus via key press as quickly and accurately as possible.

Participants completed the ANT, as described previously (Balter et al. 2019) and outlined in Figure 1. The ANT assesses three networks of attention: alerting, orienting, and executive attention. Participants completed six experimental blocks of 48 trials each. Every trial started with the presentation of a central fixation cross followed by a jittered interval of 400–500 ms. Next, one of three cue conditions was presented: No Cue, Double Cue, or Spatial Cue. In the No Cue condition, the screen with the fixation cross remained until target presentation; no information was provided about the target. In the Double Cue condition, an asterisk was presented on each side of the fixation cross to indicate when the target would appear. Finally in the Spatial Cue condition, an asterisk was presented to the left or right of the fixation cross, on the same side where the target would appear, thus indicating when and where the target would appear.

At 1.2 s after cue onset, the target array appeared: an arrow (the target stimulus) presented in the center of four similar arrows (flankers) aligned vertically on either the left or right side of the fixation cross. The flanker arrows were presented either in the same direction (congruent) or opposite direction (incongruent) as the target.

Participants were asked to report the direction of the central target stimulus as quickly and accurately as possible by pressing on the keyboard with their dominant hand. The “k” key signified an up direction of the target, whereas the “m” key signified a down direction. The target array disappeared upon the participant response or within 2000 ms, whichever occurred first.

Each cue condition was presented in pseudorandom order 16 times within each block of trials. Each combination of target array location (left or right), flanker direction (congruent or incongruent), and cue condition (no cue, double cue, spatial cue) was equally likely to occur within any given block of trials. For each experimental trial, the cue condition, target condition, accuracy (correct, incorrect), and reaction time (RT) were recorded.

### 2.5 Data Processing and Analysis

EEG data were processed offline using MATLAB v2016b toolboxes Fieldtrip (Oostenveld et al. 2011) and EEGLAB (Delorme and Makeig 2004).

#### 2.5.1 EEG Preprocessing

Trials were excluded if (1) participants gave incorrect responses, (2) RT < 150 ms or > 1500 ms, or (3) RT > 3 SDs from the mean for the relevant cue and target array combination. Continuous EEG data recorded during the ANT were then epoched into trials time‐locked to cue onset. Epochs started 2000 ms prior to cue onset and ended 2000 ms after cue onset. Data were high‐pass filtered at 0.1 Hz.

For ANT and resting‐state EEG data, semi‐automated artifact rejection was conducted using Fieldtrip followed by visual inspection for removal of muscle artifacts and noisy channels. Independent components analysis (ICA) and visual inspection were performed to remove any further ocular artifacts. Next, previously removed EEG channels were interpolated and all EEG data was average referenced.

#### 2.5.2 Spectral Power Analysis of Resting‐State Data

Cleaned resting‐state data were sliced into 2 s epochs in Fieldtrip. Power spectra were calculated using the Hanning‐windowed Fast Fourier Transform. Eight broadband EEG power spectra (1–100 Hz) were calculated from each participant: pre‐versus post‐intervention recordings (where the intervention was either saline or the typhoid vaccine) as well as both eyes‐closed and eyes‐open recordings. Frequency bands of interest were defined as delta (1–4 Hz), theta (4–8 Hz), alpha (8–13 Hz), and beta (15–40 Hz). The post minus pre‐power spectrum difference was compared via uncorrected dependent‐samples *t*‐test in each frequency band to assess topographical changes. Furthermore, the post‐intervention minus pre‐intervention subtraction was used to compare between placebo and vaccine, first via Fieldtrip's cluster‐based permutation procedure with 5000 permutations to acquire the Monte Carlo estimate of the permutation *p*‐value and cluster level test statistic. However, as no significant clusters were detected, we opted for independent‐samples *t*‐tests to assess topographical differences in each frequency band across interventions. Given a lack of significant findings, the uncorrected results (*p* < 0.05) are presented.

#### 2.5.3 Time–Frequency Analysis

Time–frequency analysis was conducted as described previously (Balter et al. 2019) and reiterated here. Sliding Hanning tapers were used to generate time–frequency representations of power per trial, such that each frequency of interest had an adaptive time window of five cycles (Δ*T* = 5/*f*). Data at trial edges were mirrored to allow for spectral power calculations.

The “alerting” time–frequency representation was assessed by subtracting no cue trials from double cue trials from –1 to 2 s relative to cue presentation. The “executive control” time–frequency representation was assessed by subtracting the congruent flanker trials from the incongruent flankers from –1 to 2 s relative to cue presentation; though the period of interest was a few hundred milliseconds (1.5–1.7 s) after target presentation, which occurred at 1.2 s. For both alerting and executive control time–frequency analyses, average power modulations from the multiple trials were assessed as an average across all electrodes prior to generating topographies of the relevant times and frequencies of interest in both postvaccine recordings and postplacebo recordings. For the alerting comparison, the alpha power at 200–400 ms postcue onset was topographically analyzed. For the executive control comparison, the theta power (4–8 Hz) at 300–500 ms was topographically analyzed. Both time–frequency and topographic differences between vaccine and placebo were analyzed using a cluster‐based permutation procedure in Fieldtrip. 5000 permutations were performed to acquire the Monte Carlo estimate of the permutation *p*‐value and cluster‐level test statistic.

“Orienting” was assessed using the alpha lateralization index (ALI) for left and right cues for early (200–500 ms postcue) and late (500–800 ms postcue) intervals. Posterior channels P3, P5, P7, P05, and P07 comprised the left ROI, and P4, P6, P8, P06, P08 comprised the right ROI. Therefore, four ALIs (left cue/late interval, left cue/early interval, right cue/late interval, right cue/early interval) were calculated in total, using Equations 1 and 2 (Haegens, Händel, and Jensen 2011). The four ALIs were assessed using a group by intervention two‐way ANOVA. Bonferroni was used to correct *p*‐values for multiple tests. The topographies of alpha power in response to left and right cues in both early (200–500 ms) and late (500–800 ms) times following cue presentation were presented.

(1) $$\;ALI_{left\;cue}=\frac{\alpha \;power\frac{Left\;ROI}{Right\;cue}-\;\alpha \;power\frac{Right\;ROI}{Right\;cue}}{\alpha \;power\frac{Left\;ROI}{Right\;cue}+\alpha \;power\frac{Right\;ROI}{Right\;cue}},\;$$

(2) $$\;ALI_{right\;cue}=\frac{\alpha \;power\frac{Left\;ROI}{Left\;cue}-\;\alpha \;power\frac{Right\;ROI}{Left\;cue}}{\alpha \;power\frac{Left\;ROI}{Left\;cue}+\alpha \;power\frac{Right\;ROI}{Left\;cue}}.\;$$

#### 2.5.4 Behavioral Data Analysis

Average RT and average accuracy were computed for each cue and target condition.

Several measures of attention network efficiency were computed using the RTs. “Alerting efficiency” was calculated as the difference between double cue and no cue trials (no cue RT—double cue RT). “Orienting efficiency” was calculated as the difference between spatial cue and double cue trials (double cue RT—spatial cue RT). “Executive control efficiency” was calculated as the difference between congruent and incongruent flanker trials (incongruent RT—congruent RT). Finally, the average RT across all conditions was computed as a measure of “psychomotor speed.”

Statistical analysis was performed using SAS 9.4 (SAS Institute, Cary NC) unless stated otherwise. Statistical analysis of the behavioral data (RT and accuracy) was conducted using *proc mixed* to implement a linear mixed model approach including group, period, treatment, and condition as fixed factors, and patient (nested in group) as a random factor. Mixed models were adjusted for baseline and the results were corrected for multiple comparisons using Bonferroni. Correlations between inflammatory markers and EEG were conducted using Spearman's correlation coefficient (*r_s_*). Results were considered statistically significant if *p* < 0.05.

## 3 Results

### 3.1 Data Quality

Exclusions are shown in Figure S1. Data from two participants on the vaccine day were excluded from analysis due to pregnancy (*n* = 1) and consumption of medication (*n* = 1) during washout. ANT data were excluded from another participant on the placebo day due to technical error (incorrect stimulus codes). Further ANT recordings were excluded from analysis due to technical error (disconnection between keyboard and computer) preplacebo (*n* = 1) and postplacebo (*n* = 1). The 72 remaining ANT and 76 resting‐state recordings were analyzed. The average number of ocular ICA components removed per participant was 2 in the ANT (SD = 0.93), 1 in the resting state with closed eyes (SD = 1.10), and 1 in the resting state with open eyes (SD = 0.74). Although the number of ICA components removed is lower than the number removed by Balter et al. (2019), the EEG data appeared clean upon visual inspection. Furthermore, participants were monitored throughout the EEG recordings and there were no issues with motion observed. For clean data acquired in a lab environment, preprocessing has been shown to have little effect on data quality (Delorme 2023).

### 3.2 ANT Behavioral Results

**FIGURE 2:**
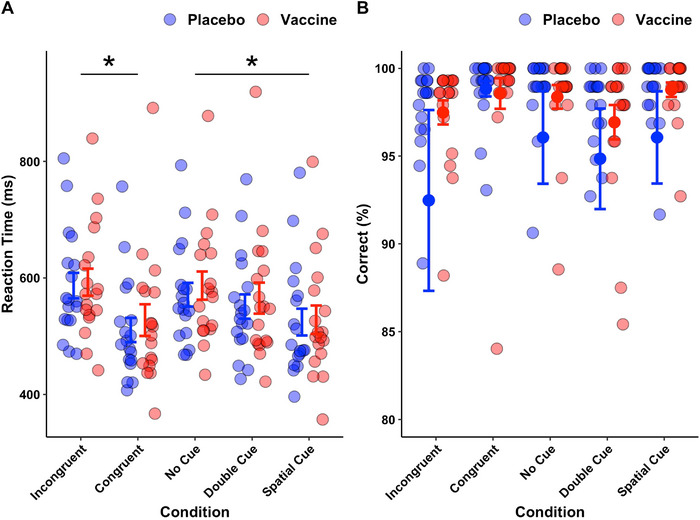
(A) Average RTs and (B) average accuracy (% correct responses) in each target flanker and cue condition following placebo (blue) and vaccine (red). Points indicate mean and error bars represent standard error. * indicates statistical significance *p* < 0.05. RT = reaction time. Participants had significantly faster reaction times in the congruent flanker condition compared to incongruent flankers, *p* < 0.0001. Participants had significantly faster reaction times in the spatial cue condition compared to the no cue condition, *p* = 0.005. There were no significant effects of intervention on RTs. There were no significant effects on accuracy.

There were no significant interactions nor main effects of intervention (placebo/vaccine) on any of the behavioral measures. However, there were significant effects of period and cue condition on RT (Figure 2A). Participants had faster reaction times in the second period (540.10 ± 11.84 ms) compared to the first period (559.48 ± 11.80 ms); a difference of 19.39 ms (95% CI: 12.84, 25.94, *p* < 0.0001). Participants had significantly faster reaction times in the congruent flanker condition (531.07 ± 12.24 ms) compared to incongruent flankers (568.51 ± 12.20 ms), a difference of 37.43 ms (95% CI: 25.59, 49.28, *p* < 0.0001). Participants had slightly faster reaction times in the spatial cue condition (538.15 ± 12.23 ms) compared to the double cue condition (552.12 ± 12.07 ms), a difference of 13.97 ms (95% CI: 3.89, 24.04, *p* = 0.07). Reaction times were significantly faster in the spatial cue compared to the no cue condition (559.10 ± 12.18 ms), a difference of 20.95 ms (95% CI: 9.30, 32.59, *p* = 0.005).

Efficiency in the vaccine condition (168.87 ± 3.37 ms) was similar to the placebo (170.04 ± 3.35 ms) condition, a difference of 1.18 ms (95% CI: –10.60, 8.25, *p* = 0.81). There was no significant interaction between intervention and efficiency (*p* = 0.27). Efficiency of alerting (139.56 ± 8.01 ms) was slightly faster than the efficiency of executive control (155.96 ± 6.64 ms), a difference of 16.40 ms (95% CI: 2.66, 30.15, *p* = 0.12, uncorrected *p* = 0.02). Efficiency of orienting (143.68 ± 7.71 ms) was not significantly different to alerting (*p* > 0.99) nor executive control (*p* = 0.45). Efficiency in each condition is shown in Figure S2. Alerting efficiency correlated negatively with log IL‐6 in the placebo condition (*r_s_* = –0.599, uncorrected *p* = 0.009).

There were no significant interaction nor main effects observed on accuracy (Figure 2B). Accuracy in the placebo (96.91 ± 0.29 %) and vaccine (96.81 ± 0.29 %) conditions was similar, a difference of only 0.10 % (95% CI: –0.67, 0.47, *p* = 0.731). Accuracy in the incongruent flanker (96.76 ± 0.38 %) and congruent flanker (96.95 ± 0.38 %) conditions were comparable, a difference of 0.20 % (95% CI: –1.08, 0.69, *p* > 0.99). Similarly, there were no significant differences between the no cue (97.30 ± 0.38 %), double cue (96.41 ± 0.38 %), and spatial cue (96.86 ± 0.38 %) conditions, all *p*s > 0.99. Accuracy in the double cue condition following the vaccine was weakly correlated with log IL‐6 (*r_s_* = 0.581, uncorrected *p* = 0.011).

### 3.3 EEG

**FIGURE 3:**
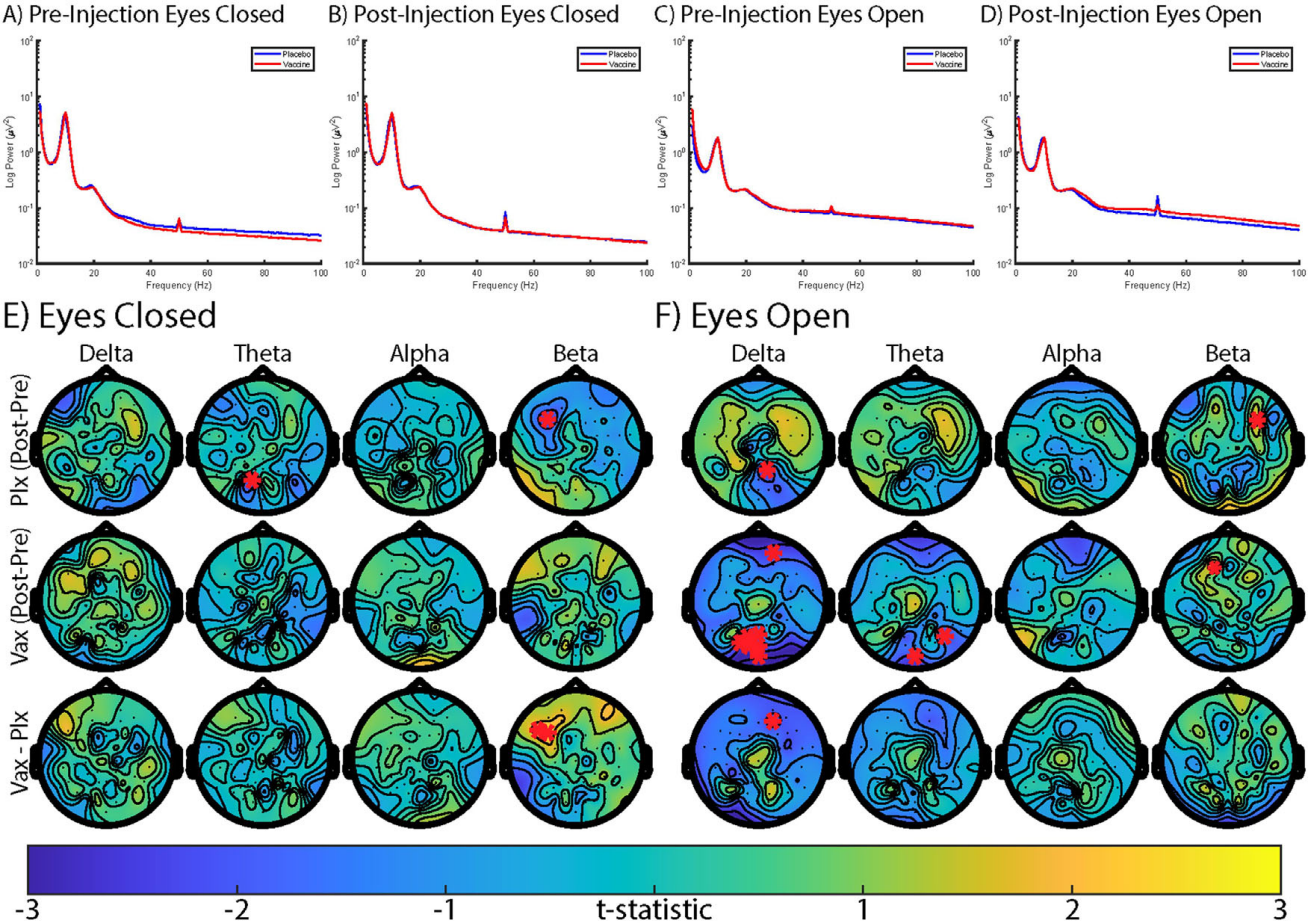
EEG resting‐state recordings during (A) preintervention eyes‐closed, (B) postintervention eyes‐closed, (C) preintervention eyes‐open, (D) postintervention eyes‐open. Red indicates vaccine and blue indicates saline intervention. (E) is eyes‐closed and (F) is the eyes‐open scalp topography of delta (1–4 Hz), theta (4–8 Hz), alpha (8–13 Hz), and beta (15–40 Hz) power. The first two rows of (E) and (F) denote the uncorrected, paired post‐intervention minus pre‐Balintervention *t* parametric map. The third row denotes an uncorrected, unpaired *t* parametric map comparing between the Vaccine (Vax) and Placebo (Plx) group. Red asterisks *indicate significant (*p* < 0.05) differences from uncorrected comparisons.

Spectral analysis of uncorrected resting‐state data (Figure 3) show few significant changes. Broadband spectral analysis shows similar spectral curves between vaccine and placebo in all four recordings (Figure 3A–D). The topographies of the eyes‐closed scans show a significant decrease in occipital theta and in left‐frontal beta when comparing post minus pre placebo (Figure 3E). A significant channel‐level difference between interventions was observed in the left‐frontal beta band during eyes‐closed recordings, though this appeared to be largely driven by a significant placebo decrease. The eyes‐open scan topographies show a decrease in occipital delta and increase in right‐frontal beta when comparing post minus pre placebo (Figure 3F). When comparing post minus pre vaccine, decreases in frontal and occipital delta, occipital theta, and an increase in left‐frontal beta were observed. In observing the topographical changes between the interventions a decrease in delta power was observed in frontal delta, largely driven by the decrease in the vaccine condition.

**FIGURE 4:**
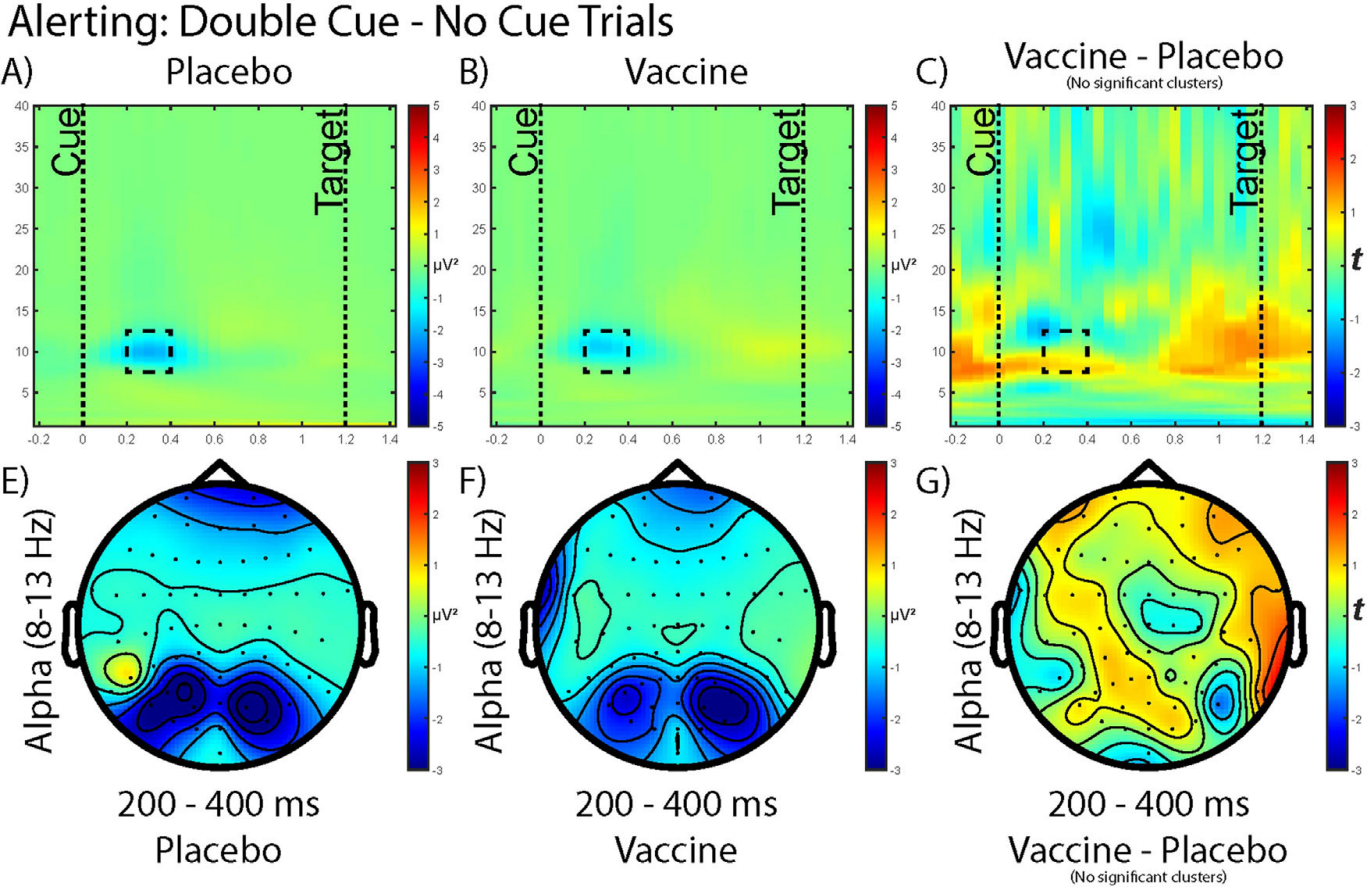
Time–frequency analysis of the alerting dimension of the attention network task after (A) placebo intervention, (B) typhoid vaccine intervention, and (C) comparing between the vaccine and placebo response. The box (with the dashed line) indicates where the subsequent alpha topographies are extracted from. The topography of the alpha (8–13 Hz) power at 200–400 ms is mapped out in (E) after placebo, (F) after vaccine, and (G) comparing between vaccine and placebo. The cue was initiated at 0 s and the target presented at 1.2 s. Cluster‐based permutation comparisons (with 5000 iterations) showed no statistically significant clusters.

Time–frequency analysis of the alerting (double cue—no cue, Figure 4A–G) trials did not reveal any significant clusters from the cluster‐based permutation procedure. However, a slight decrease in alpha power was observed at 200–400 ms postcue presentation and topographical analysis showed a decreased occipital alpha topography, though comparing this topography between vaccine and placebo did not yield significant differences. The scalp topography of alerting‐related alpha activity indicates occipital alpha suppression in both the placebo and vaccine conditions. We observed a trend toward a significant association between alerting alpha power and log IL‐6 in both placebo (*r_s_* = –0.455, uncorrected *p* = 0.057) and vaccine (*r_s_* = –0.459, uncorrected *p* = 0.055) conditions. Scatterplots of these data are shown in Figure S3.

**FIGURE 5:**
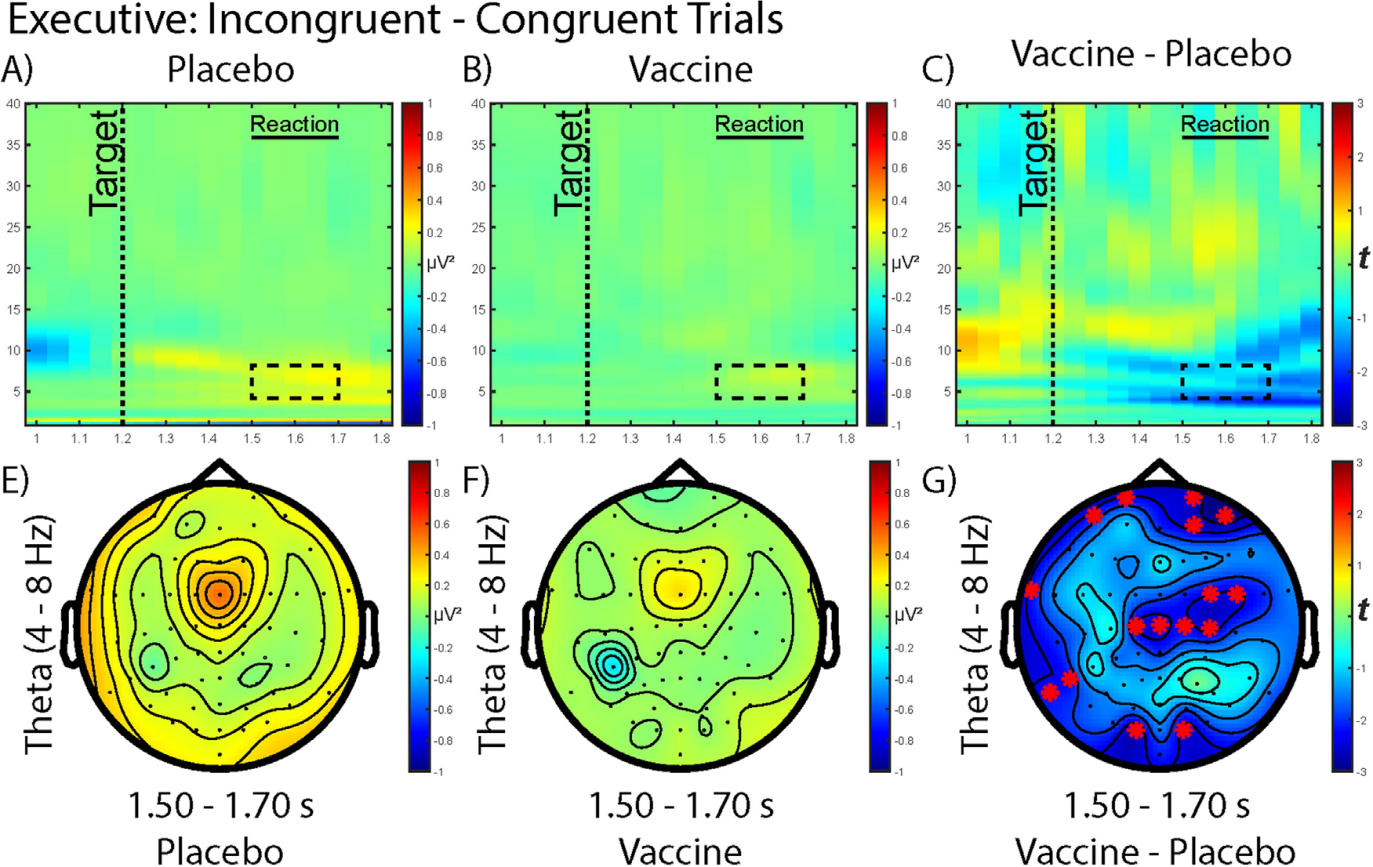
Time–frequency analysis of the executive control of the attention network task after (A) placebo intervention, (B) typhoid vaccine intervention, and (C) comparing between the vaccine and placebo response. The box (with the dashed line) indicates where the subsequent theta topographies are extracted from. The target was presented at 1.2 s and participants’ reaction time were approximately at 1.5 s. The topography of the theta (4–8 Hz) power at 1.5–1.7 s is mapped out in (E) after placebo, (F) after vaccine, and (G) comparing between vaccine and placebo. Cluster‐based permutation comparisons (with 5000 iterations) showed red asterisks * indicating significant (*p* < 0.05) differences in the channels between interventions.

Time–frequency analyses of the executive control (incongruent—congruent, Figure 5A–G) trials did not yield any significant clusters. However, the postvaccine time–frequency representation exhibited muted spectral power changes compared to the postplacebo recording. The vaccine topography of theta (4–8 Hz) power at 300–500 ms posttarget presentation (or, 1.5–1.70 s postcue onset) showed slight decreases to theta midline central power compared to the placebo topography. The comparison of vaccine minus placebo showed multiple channels with significant differences (after cluster correction) across the frontal, mid‐line, and occipital regions likely driven by the decreased theta in the vaccine condition.

**FIGURE 6:**
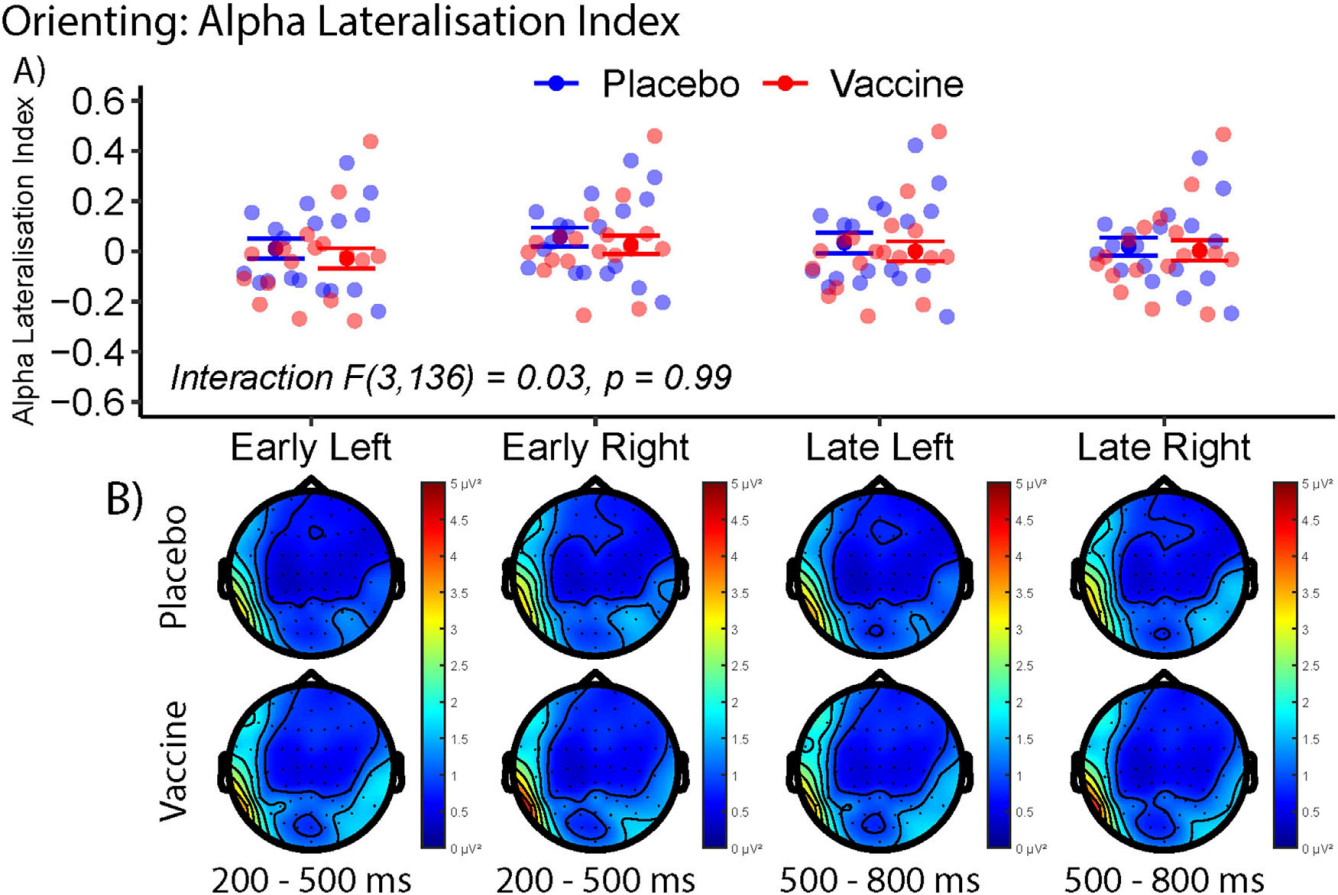
Alpha lateralization index (ALI) as an indicator for orienting spatial attention. (A) The ALI of placebo (blue) and typhoid vaccine (red) in response to left and right cues in early (200–500 ms) and late (500–800 ms) periods postcue presentation. A two‐way interaction ANOVA showed no significant differences. (B) The corresponding topography of alpha (8–13 Hz) power in postvaccine and postplacebo recordings.

In identifying the effect of intervention on alpha lateralization index (ALI) and orienting postcue, no significant differences were observed between the interventions (Figure 6A, B). There was no significant interaction observed between the ALI and intervention condition (*F* = 0.03, *p* = 0.99) and there were also no significant interactions between intervention condition and cue or time. No significant correlations between the ALI and overall RT nor between ALI and the IL‐6 response were observed.

## 4 Discussion

### 4.1 Behavioral Results

Behavioral and neurophysiological results were broadly similar in both inflammation and placebo conditions. Behavioral responses to the ANT were in line with previous works (Balter et al. 2019; Fan et al. 2002; Redick and Engle 2006; Neuhaus et al. 2010; Fan et al. 2009), indicating that the task was completed by participants appropriately. Regarding cue condition, the speed of RTs is usually fastest in spatial cue trials, followed by double cue, then no cue trials (Figure 2A). This pattern of RTs makes sense given the amount of information provided by each condition; conditions that offer more information on the upcoming target confer faster RTs. Regarding target flankers, congruent trials are usually faster than incongruent trials (Figure 2A). Incongruent trials require the identification of a mismatch between the target and flanker arrows, perhaps resulting in longer RTs compared to congruent trials where no mismatch exists. The absence of inflammation effects suggests that the information provided by cues and target flankers was used equally in vaccine and placebo conditions. RTs in the second period were faster than the first period, likely corresponding to a practice effect; participants were faster at completing the task on their second study visit than the first.

The absence of significant treatment effects on behavioral performance is consistent with prior studies of acute inflammation on attention tasks (Brydon et al. 2008). Tasks involving cognitive processing, attention, and executive control, yield similar levels of accuracy in both inflammation and placebo conditions (Bollen et al. 2017). Administration of endotoxin has previously been suggested to affect motivation, without any noticeable effect on performance. Participants have self‐reported decreases in attention during cognitive tasks though their performance is maintained, perhaps indicating an adaptive response to immune challenge.

### 4.2 Resting‐State Spectral Power

An inflammation‐mediated decrease was observed in delta power in the eyes‐open scan, whereas a relative increase was observed in beta power in the eyes‐closed scan (Figure 3). van den Boogaard et al. (2010) observed increases in relative and absolute band power for alpha and beta activity in frontocentral and parieto‐occipital regions following LPS, indicating a higher state of alertness conferred by endotoxin. However, these changes were not observed in the present results (Figure 3), possibly due to the milder effects of the typhoid vaccine relative to LPS (Schedlowski, Engler, and Grigoleit 2014). Van den Boogaard et al. also found a significant association between increased cortisol and the increased peak frequency in the occipital electrodes. Endotoxin‐induced cortisol secretion likely results in higher alertness, whereas placebo is unlikely to cause a stress hormone induced alertness response. A relative decrease in beta following placebo is consistent with the absence of a cortisol mediated stress response.

Decreased frontal‐delta power in the eyes‐open resting state was likely driven by decreased delta in the vaccine condition. Reduced frontal‐delta power was found recently in a study of long‐COVID‐19 patients compared to healthy controls (Ortelli et al. 2023). Chronic inflammation has been implicated in long‐COVID pathology (Yin et al. 2024). The decrease in delta power observed following vaccination is thus in accordance with the findings from long‐COVID patients, given the inflammatory etiology of both states. A reduction in frontal‐delta power has also previously been linked to psychological pain in patients with major depressive disorder (Meerwijk, Ford, and Weiss 2015), a condition with a posited inflammatory basis (Raison, Capuron, and Miller 2006). Anecdotal reports from our participants suggested that typhoid vaccine caused pain in the arm at the site of injection. Prior studies of typhoid vaccine additionally show worsened mood several hours following injection (Strike, Wardle, and Steptoe 2004). In light of this prior research, our findings suggest that reduced frontal‐delta power may occur due to an inflammation‐mediated unpleasant internal state.

### 4.3 ANT Time–Frequency Analysis

Balter et al. (2019) found greater suppression of alpha power 200–300 ms following cue onset in the vaccine condition compared to placebo (Balter et al. 2019). In this trial, suppression of alpha power was thought to indicate the greater cognitive exertion required to maintain cognitive performance in an inflammatory state. Previous work demonstrated that alpha power suppression increases with task demands (Fink et al. 2005). Thus, the extent of alpha power suppression may correspond to the level of cognitive effort required by a cognitive task. Balter et al. (2019) found suppression of alpha power specifically during the alerting phase of the ANT. We found a decrease in occipital alpha, though no significant difference was observed between the vaccine and placebo. The scalp topography appears to replicate that of Balter et al. (2019); however, the alpha suppression in the vaccine condition appears less intense. The pattern of our results is similar; however, our findings did not reach the threshold for significance.

We also calculated the alpha lateralization index (ALI) and found no significant relationship between ALI and the intervention condition. The EEG time–frequency assessment of the ALI represents the orienting attentional network, where information is selected from the double and spatial cue trials prior to response. The absence of significant effects on the ALI suggests inflammation does not affect the orienting aspect of attention, which is consistent with the findings from Balter et al. (2019). Spatial information from cues is used similarly and has comparable effects on the posterior EEG regardless of inflammatory state.

For the executive control aspect of attention, we found a significant decrease in theta activity with inflammation. The scalp topography of frontal theta executive control in the placebo condition appears similar to the presentation in Balter et al. (2019). Greater frontal midline theta is expected in situations requiring more cognitive control. For example, coordination of inhibition during the Stroop task increases frontal midline theta (Eschmann, Bader, and Mecklinger 2018). A study that utilized the ANT to assess postacute sequelae of COVID‐19 (long‐COVID) showed selective executive control impairment, whereas orienting and alerting networks were unaffected (Lamontagne et al. 2021). Unfortunately the study is limited to behavioral results from the ANT and did not record EEG. Chronic inflammatory conditions like long‐COVID likely affect the behavior of individuals to a far greater extent than the typhoid vaccine injection. While both are associated with a systemic inflammatory reaction, long‐COVID may elicit severe symptoms including debilitating fatigue and brain fog. Typhoid vaccine only elicits very mild symptoms for participants, thus the participants in our study did not experience any performance decrement following vaccine administration. However, the relative decrease in theta following vaccine may suggest a compensatory mechanism at play that enables maintenance of performance in the inflammation condition.

Generally, our results showed a trend toward those presented in previous work, though our results did not reach the threshold for significance despite similar study sizes (*N* = 20). The absence of significance in our study may be linked to the level of inflammation. The average IL‐6 concentration in the present study was 1.6 pg/mL, compared to 5.1 pg/mL in the previous trial (Balter et al., 2019). Moreover, the authors found log IL‐6 was significantly negatively associated with alerting‐related alpha power postvaccine. Our results trended toward an association between log IL‐6 with alerting‐related alpha power postvaccine but again did not reach significance, perhaps indicating that the concentration of IL‐6 was too low to show a significant relationship with alpha power. In our study, the vaccine did not elicit an inflammatory response strong enough to yield a significant effect on alpha power indicative of greater cognitive exertion.

Additionally, differences between our samples may have led to less significant findings in the present study. Equal numbers (10 each) of males and females participated in the current study, whereas Balter et al. (2019) used a sample of 20 males. Sex differences have previously been reported for resting‐state (Cave and Barry 2021) and task‐based EEG (Davidson et al. 1976). It may be that inflammation‐induced alpha power suppression is stronger (or more consistent) in males compared to females. It is worth noting that the average age of participants in the present study is 34 years, approximately ten years older than the average age of participants in the earlier study. Aging can also influence the EEG signal (Dustman et al. 1985), though previous work usually focuses on age gaps that are much larger than those observed here.

### 4.4 Strengths and Limitations

The present study is one of few to examine the effect of an acute inflammatory stimulus on resting‐state EEG in healthy volunteers during wakefulness. The inclusion of a measure of symptomatology would be useful in the future to assess any observed changes in the body felt by participants posttreatment. The present study used a balanced sample of males and females, whereas previous experimental EEG studies used all male samples to assess the effects of endotoxin (van den Boogaard et al. 2010; Haack et al. 2001; Korth et al. 1996; Pollmacher et al. 1993; Mullington et al. 2000). Although this limits our ability to compare to previous studies, it also increases the study's relevance to the general population. Oscillations in EEG are noted among female participants during the menstrual cycle (Solis‐Ortiz et al. 1994) though we did not assess this in the current study. Finally, a very low level of inflammation was induced by typhoid vaccine in the present study, which likely reduced possible effects of inflammation on the brain. Due to the exploratory nature of the resting‐state analysis, the resting‐state EEG results were not corrected for comparisons and thus should be interpreted cautiously. This study may be helpful to inform future work on inflammation‐mediated alterations to human EEG.

## 5 Conclusion

The present study found some evidence of inflammatory‐mediated changes to the alerting attention network; however, our findings did not cross the significance threshold. The absence of significant findings may be due to the low level of inflammation induced by the typhoid vaccine, such that the level of inflammation was not sufficient to provoke a neurophysiological response. Further experimental studies are needed to fully elucidate the mechanism underlying acute inflammatory effects on the attention network. Moreover, identifying inflammatory‐mediated changes to resting‐state EEG could provide useful clinical applications for inflammatory conditions; however, current work has not established reliable biomarkers.

## Author Contributions

**Julia Plank**: Investigation; formal analysis; writing—original draft; visualization. **Joseph Chen**: Formal analysis; software; visualization, writing—review and editing. **Frederick Sundram**: Conceptualization; resources. **Nicholas Hoeh**: Resources. **Suresh Muthukumaraswamy**: Methodology; conceptualization; resources; software; supervision; writing—review and editing. **Joanne Lin**: Conceptualization; methodology; funding acquisition; supervision.

## Ethics Statement

All procedures performed in studies involving human participants were in accordance with the ethical standards of the institutional and/or national research committee and with the 1964 Helsinki declaration and its later amendments or comparable ethical standards.

## Informed Consent

Informed consent was obtained from all individual participants included in the study.

## Conflicts of Interest

The authors declare no conflicts of interest.

### Peer Review

The peer review history for this article is available at https://publons.com/publon/10.1002/brb3.70249

## Supporting information



Supporting Information

## Data Availability

The data that support the findings of this study are available from the corresponding author upon reasonable request.
