# Supplementary material for: The Effects of Neuroinflammation Induced by Typhoid Vaccine on Resting and Task‐Based Electroencephalography
Source: Brain Behav. 2024 Dec 31;15(1):e70249. doi: 10.1002/brb3.70249 (PMC11688119; doi:10.1002/brb3.70249)
Supplement: Supplementary file 1 — Supporting Information [file BRB3-15-e70249-s001.docx]

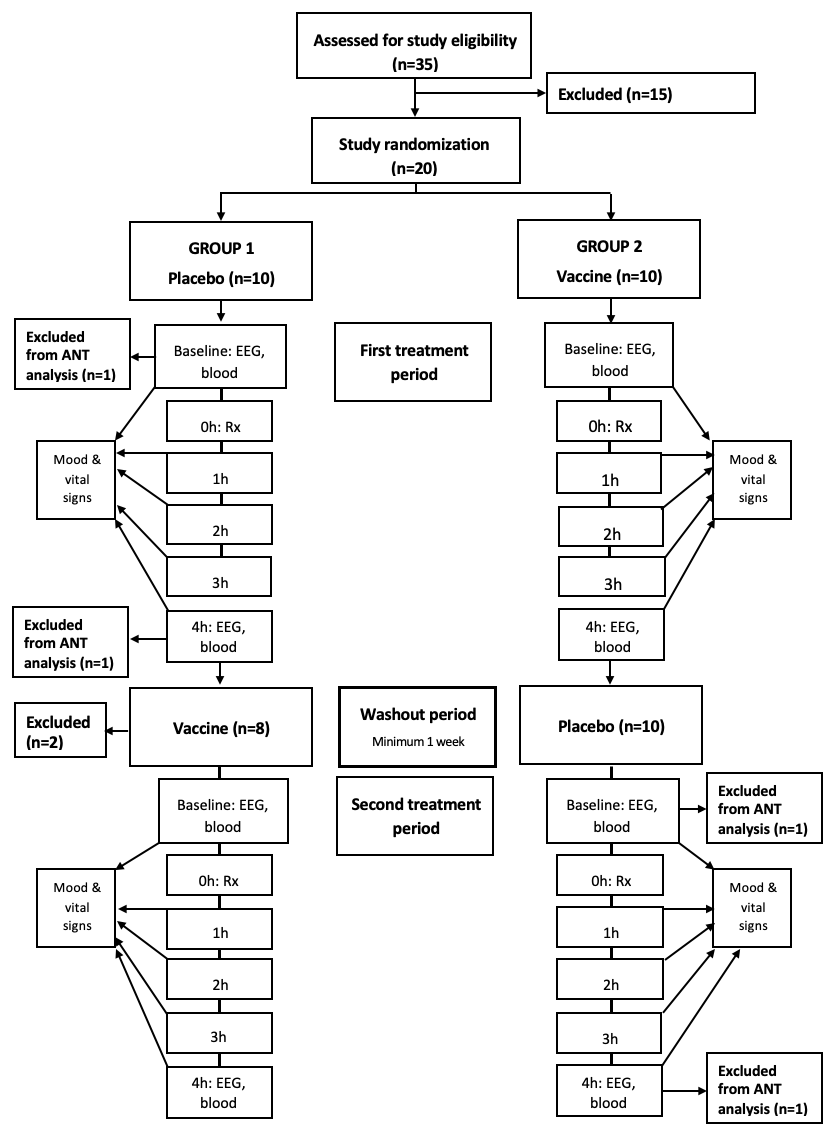


**Figure 1. Flow of study including exclusions.** Rx = treatment administration (placebo/vaccine).

**
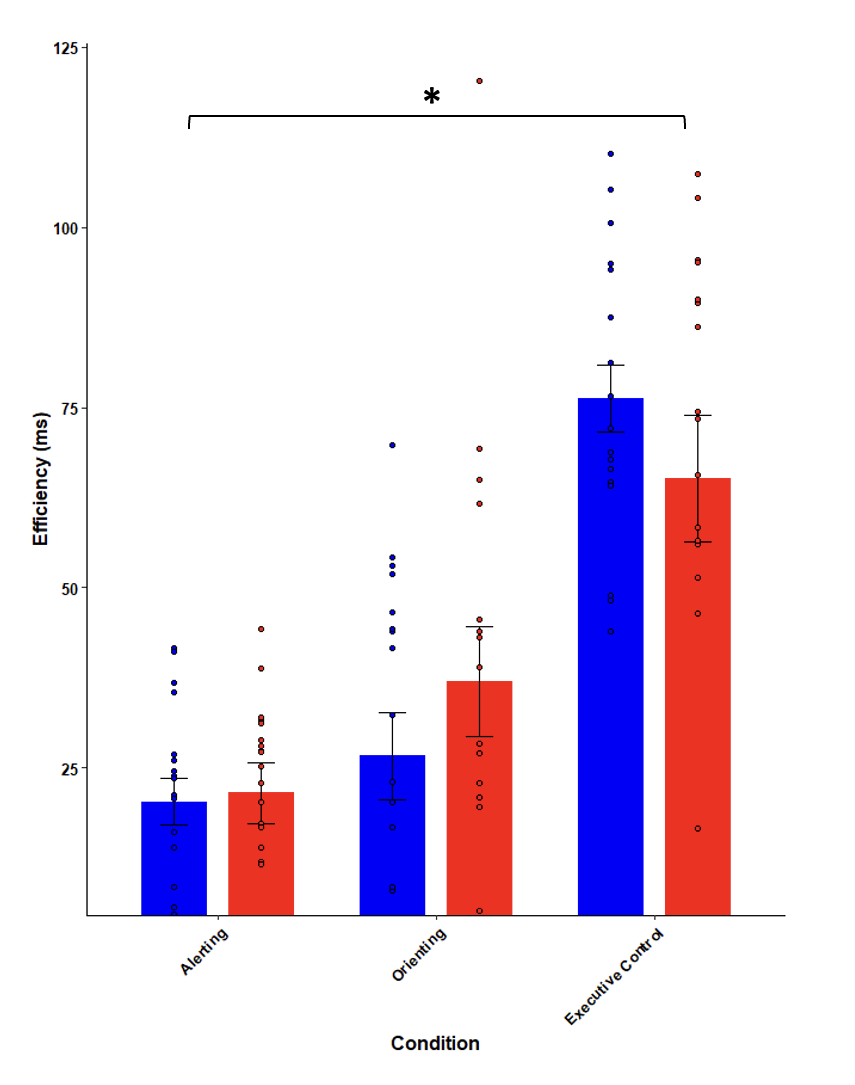
**

**Figure 2. Efficiency of alerting, orienting, and executive control and following placebo (blue) and vaccine (red).**

Error bars represent standard error. * indicates statistical significance uncorrected *p*<0.05. Efficiency of alerting was significantly faster than the efficiency of executive control, *p*=0.020.

*Alerting efficiency = difference in RT between no cue and double cue trials; executive control efficiency = difference in RT between incongruent flanker and congruent flanker trials; orienting efficiency = difference in RT between double cue and spatial cue trials; RT = reaction time.*

**
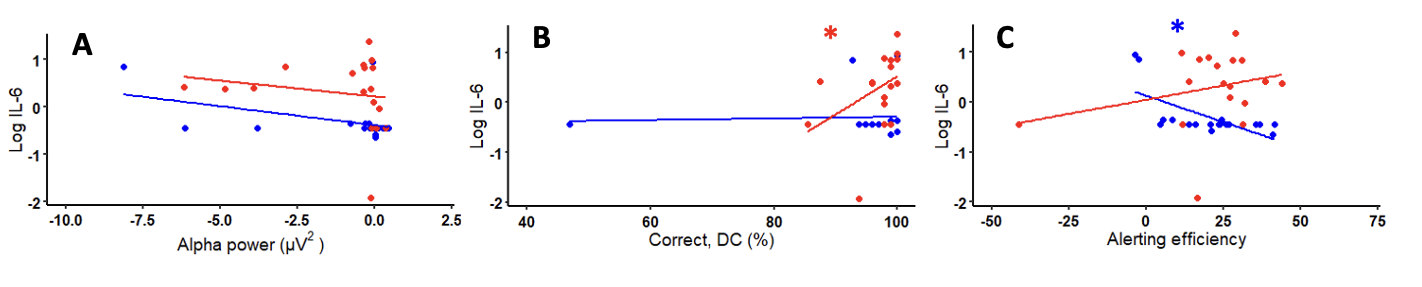
**

**Figure 3. Scatterplots of inflammatory markers versus ANT measures following placebo (blue) and vaccine (red).**

Significant correlations (uncorrected *p*<0.05) are marked with *.

Description of plots: A) alerting alpha power and log IL-6 post-placebo (r_s_ = –0.455, *p* = 0.057) and post-vaccine (r_s_ = –0.459, *p* = 0.055); B) post-vaccine accuracy in double cue condition and log IL-6 (r_s_ = 0.581, *p* = 0.011); C) post-placebo log IL-6 and alerting efficiency (r_s_ = –0.599, *p* = 0.009).

*Alerting efficiency = difference in RT between no cue and double cue trials; ALI = alpha lateralisation index; ANT = attention network test; DC = double cue; executive efficiency = difference in RT between incongruent flanker and congruent flanker trials; orienting efficiency = difference in RT between double cue and spatial cue trials; RT = reaction time.*
